# Study on the Mechanism of a Side Coupling Reaction during the Living Anionic Copolymerization of Styrene and 1-(Ethoxydimethylsilyphenyl)-1-phenylethylene (DPE-SiOEt)

**DOI:** 10.3390/polym9050171

**Published:** 2017-05-11

**Authors:** Pibo Liu, Hongwei Ma, Heyu Shen, Li Han, Shuang Chang, Long Zang, Yiyu Bian, Yu Bai, Yang Li

**Affiliations:** State Key Laboratory of Fine Chemicals, Liaoning Key Laboratory of Polymer Science and Engineering, Department of Polymer Science and Engineering, School of Chemical Engineering, Dalian University of Technology, Dalian 116024, China; ipoy@mail.dlut.edu.cn (P.L.); shy920218@mail.dlut.edu.cn (H.S.); lili88882008@126.com (L.H.); dlmuchang@163.com (S.C.); 201549043@mail.dlut.edu.cn (L.Z.); 201449053@mail.dlut.edu.cn (Y.B.); 201549024@mail.dlut.edu.cn (Y.B.)

**Keywords:** living anionic polymerization, 1,1-Diphenylethylene-SiOEt, functionalized polymers, side reaction

## Abstract

A 1,1-diphenylethylene (DPE) derivative with an alkoxysilyl group (DPE-SiOEt) was synthesized. It was end-capped with poly(styryl)lithium (PSLi) and then copolymerized with styrene via living anionic polymerization (LAP) in a non-polar solvent at room temperature. The observed side coupling reaction was carefully investigated by end-capping the polymer. Changes in molecular weight support the plausibility of a mechanism involving living anionic species (PSLi or lithiated DPE-end-capped polystyrene, PSDLi) and the alkoxysilyl groups. Through a series of copolymerizations with different feed ratios, the kinetics of the side coupling reaction were also studied. The results showed that the side reactions could be controlled using an excess feed of DPE-SiOEt, a potentially useful strategy for the synthesis and application of well-defined alkoxysilyl-functionalized polymers via LAP.

## 1. Introduction

Living anionic polymerization (LAP) enables the synthesis of well-defined polymers with controllable molecular weights and narrow distribution, and thus has important applications in the synthesis of functionalized [1,2,3,4] and topological polymers [5,6]. When certain functional groups are grafted onto monomers, the highly active living species undergoing chain propagation could be terminated or transferred during polymerization, causing the molecular weight and molecular weight distribution of the resulting polymer to be uncontrolled [7,8]. Polymer chemists have investigated these side reactions between certain functional groups and living anionic centers and developed some strategies to prevent them [9,10,11]. For example, lowering the polymerization temperature [9], protecting the functional groups [9], and reducing the activity of the living anionic species [10,11] have been employed to stabilize the reactive center and avoid unwanted side reactions. However, these reactions are still the primary problem for chemists attempting to develop functionalized polymers. Therefore, more investigations are needed to design new functional monomers with functional groups that can tolerate the harsh conditions of LAP and prevent side reactions during polymerization.

The use of 1,1-diphenylethylene (DPE) derivatives, which is one of the strategies used to prevent side reactions during LAP, can efficiently reduce the activity of the living species and stabilize the reactive center, due to their distinctive chemical structure. For example, synthesizing block copolymers of styrene and methyl methacrylate using DPE to avoid any possible side reactions has become a common method [12]. When the polymerization of methylmethacrylate was directly initiated by poly(styryl)lithium (PSLi), the molecular weight distribution of the copolymer broadened because of side termination reactions. However, these side reactions were effectively reduced when PSLi was end-capped with a DPE unit. DPE derivatives can also reduce side reactions between the anionic active center and certain functional groups grafted to DPE units. In a previous investigation, obvious termination was observed when the living anionic homopolymerization of amino-functionalized styrene derivative was conducted at room temperature [13]. By contrast, the living anionic copolymerization of styrene and an amino-functionalized DPE derivative (DPE-NMe_2_ or DPE-(NMe_2_)_2_) avoided the side termination reaction and maintained the narrow distribution of the product [14,15]. Based on this interesting strategy, we were inspired to use the DPE structure to introduce other functional moieties into polymer chains and avoid unexpected side reactions during living polymerization.

Because of the remarkable steric hindrance of DPE derivatives, the sequence distribution of their functional groups in polymer chains has also been determined and controlled [16,17,18,19]. However, this approach requires stable functional substitutes. For the precise introduction of chain-end vinyl groups, the end-capping of a DPE derivative with an α-methylstyryl group (DPE-ene) with PSLi was conducted in benzene at room temperature; however, the corresponding dimer was observed. Moreover, in the presence of a polar additive, the side transfer reaction clearly became predominant [7]. Thus, chain-end functionalized polymers with α-methylstyryl groups must be prepared at low temperature. In the future, side reactions will be a key obstacle to the synthesis of sequence-defined functionalized polymers, and, therefore, more functional groups will be introduced into polymer chains with controlled sequences using DPE derivatives via LAP.

Herein, a novel DPE derivative 1-(ethoxydimethylsilyphenyl)-1-phenylethylene (DPE-SiOEt), which possesses an alkoxysilyl group, was synthesized and characterized. To synthesize well-defined/alkoxysilyl end-chain-functionalized polymers via LAP, the mechanism and kinetics of the side reactions between the living species and the alkoxysilyl groups grafted to the DPE structure were investigated.

## 2. Materials and Methods 

### 2.1. Materials

Tetrahydrofuran (THF) was dried by reflux over Na-benzophenone complex and then distilled under argon. Diethoxydimethylsilane (Aldrich, Shanghai, China, 99%) was stirred over freshly ground calcium hydride under argon for over 12 h and then distilled. Methyltriphenylphosphonium bromide (Aldrich, Shanghai, China, 98%), potassium *tert*-butoxide (Aldrich, Shanghai, China, 98%), magnesium turnings for the Grignard reaction (STREM, Milford, CT, USA, 99%) and 4-bromobenzophenone (Energy Chemical, Shanghai, China, 98%) were used as received.

The purification of styrene, benzene, and THF for anionic polymerization were conducted under high vacuum conditions as described in the literature. *sec*-Butyllithium (*sec*-BuLi) was prepared using 2-chlorobutane and lithium metal in benzene under high vacuum conditions as well. The concentration of *sec*-BuLi was 0.3044 mol/L, as determined by double titration.

### 2.2. Methods

^1^H NMR (5 wt %, CDCl_3_) spectra were recorded on a Bruker Avance II 400 MHz NMR spectrometer with (CH_3_)_4_Si (tetramethylsilane, TMS) as an internal standard. Size exclusion chromatography (SEC) was performed on a Waters HPLC component system (2414 refractive index detector) at a flow rate of 1.0 mL/min in THF at 30 °C after calibration using polystyrene standards. MALDI-TOF-MS analysis was performed on a Waters MALDI micro MX mass spectrometer (Waters, Milford, CT, USA) with 2-[(2E)-3-(4-*tert*-butylphenyl)-2-methyprop-2-enylidene] malonitrile (DCTB) and sodium trifluoroacetate as dopants; details of the sample preparation are provided in a previous study [14].

**1-(4-bromophenyl)-1-phenylethylene (DPE-Br):** DPE-Br was synthesized using classic Wittig reaction conditions with 4-bromobenzophenone as the substrate under argon. Typically, methyltriphenylphosphonium bromide (219 g, 0.61 mol) and freshly distilled THF (200 mL) were added into a round bottom flask equipped with a reflux condenser and a magnetic stir bar. Potassium *tert*-butoxide (670 mL of a 1.0 M solution in THF, 0.67 mol) was then added dropwise to the reaction flask via a constant pressure funnel. The reaction mixture was stirred for 2 h at −20 °C. Then, a solution of 4-bromobenzophenone (100 g, 0.38 mmol) in dry THF (400 mL) was added dropwise to the reaction mixture via constant pressure funnel at −20 °C. The orange-brown reaction mixture was stirred overnight at −20 °C. The resultant mixture was extracted with diethyl ether three times. The ethereal layer was washed with aqueous NaHCO_3_ solution and saturated aqueous NaCl solution and then dried over MgSO_4_. After filtration, the ethereal layer was poured into hexane to precipitate triphenylphosphine oxide, and then the solution was filtrated and concentrated under reduced pressure. The product was distilled at 120 °C (4.8 Pa) to give 94 g (0.36 mol, 94%) of DPE-Br as a yellow liquid (purity 99.2% GC); 400 MHz ^1^H NMR (CDCl_3_): δ = 5.44 and 5.42 (d, ^2^*J*_H,H_ = 8.21 Hz, 2 × 1H, =CH_2_), 7.43 and 7.19 (dd, ^3^*J*_H,H_ = 8.21 Hz, 2 × 2H, Ar-H), 7.30 (m, 5H, Ar-H) ppm.

**1-(ethoxydimethylsilyphenyl)-1-phenylethylene (DPE-SiOEt):** A solution of DPE-MgBr (0.19 mol, prepared from 1-(4-bromophenyl)-1-phenylethylene (50 g, 0.19 mol) and magnesium (23 g, 0.97 mol) in dry THF (800 mL)), was added dropwise over 3 h to a solution of diethoxydimethylsilane (60 g, 0.4 mol) in dry THF (100 mL). The reaction mixture was stirred at room temperature overnight under argon. The crude product was obtained from the mixture by direct distillation and was purified carefully by fractional distillation at 120~130 °C (4 Pa) to give 28 g (0.13 mol, 52%) of DPE-SiOEt as a colorless liquid (purity 99.4% GC). 400 MHz ^1^H NMR (CDCl_3_): δ = 0.41 (s, 6 × 1H, –CH_3_), 1.20, 1.21 and 1.23 (t, ^2^*J*_H,H_ = 6.98 Hz, 3 × 1H, –CH_3_), 3.69, 3.70, 3.72 and 3.74 (q, ^2^*J*_H,H_ = 6.98 Hz, 2 × 1H, –CH_2_–), 5.49 and 5.50 (d, ^2^*J*_H,H_ = 5.20 Hz, 2 × 1H, =CH_2_), 7.37 and 7.56 (dd, ^3^*J*_H,H_ = 8.12 Hz, 2 × 2H, Ar-H), 7.34 (m, 5H, Ar-H) ppm. ^13^C NMR: 150.1, 142.6, 141.4, 137.5, 133.5, 128.4 128.3, 127.8, 127.7, 114.6, 25.8, 18.6, −1.5 ppm, as shown in Figure S1. *m*/*z* (%): 282.1448 [M]^+^, 267.1207 [M–CH_3_]^+^, 223.0948 [M–OC_3_H_7_]^+^.

**Sodium 2,3-dimethylpentan-3-olate** **(NaODP):** In a dry box, 200 mL benzene and excess sodium (Na/–OH = 10 eqv.) were added into a flask, and then, purified 2,3-dimethyl-3-pentanol (*d* = 0.829 g/mL, 0.02 mol) was added into the flask. The reaction mixture was stirred at room temperature for 7 days in the dry box. The synthesis route is shown in Figure S3 in the ESI. Finally, the excess sodium was removed by filtration, and the product concentration was determined to be 0.4037 mol/L by titration.

### 2.3. Polymerization Procedures

#### 2.3.1. End-Capping PSLi with DPE-SiOEt

First, 1.0 g (9.6 mmol) of styrene in 10 mL benzene was initiated by adding 1.6 ml of *sec*-BuLi (0.48 mmol, *c* = 0.3044 mol/L) with a designed molecular weight of 2 kg/mol at 25 °C for 4 h. An aliquot was removed from the solution for further tests, and then, 0.17 g (0.60 mmol) of DPE-SiOEt (DPE-SiOEt/PS-Li = 1.2) was added into the solution. After the solution was kept at 25 °C for 2 h, the product was precipitated using excess methanol and subsequently dissolved in toluene; this process was repeated twice, and then the product was dried to a constant weight in a vacuum oven.

#### 2.3.2. Copolymerization of Styrene and DPE-SiOEt

The copolymerization of styrene and the DPE derivative was conducted in benzene using *sec*-BuLi as the initiator in a glove box under argon. NT-1 in Table 1 is taken as an example for illustration: 15 mL of benzene and 1.00 g (3.56 mmol) of DPE-SiOEt were added to a Schlenk under argon, and then, 0.24 mL (0.3044 mol/L, 0.07 mmol) of *sec*-BuLi was added. The characteristic dark red color of the alkoxysilyl-substituted 1,1-diphenylalkyllithium anion appeared immediately. The solution was stirred for 15 min to ensure that the corresponding 1,1-diphenylalkyllithium was initiated sufficiently. Then, 0.41 mL (3.56 mmol) of styrene was added and reacted at 25 °C for 24 h. The products were precipitated with excess methanol and subsequently dissolved in toluene; the process was repeated twice until the residual DPE derivative monomer was completely removed, and then the product was dried to a constant weight in a vacuum oven.

## 3. Results and Discussion

Alkoxysilyl groups are useful tools for preparing hybrid composites by forming a bridge between an organic matrix and inorganic surface through [Si-O-Si] covalent bonds. [SiO-R-X]-type molecules are used to modify silicon or silica surfaces (SiO- is an alkoxysilyl group and -X is a halogen group) and, after further modification, can act as initiators for controlled radical polymerization (CRP) methods [20,21] or living anionic polymerization (LAP) [22]. However, LAP, even if conducted at low temperature, is unsuitable for synthesizing alkoxysilyl-functionalized polymers because of the unavoidable side reactions between the alkoxysilyl groups and the living species [18].

As mentioned above, to avoid these side reactions, DPE has been used as an end-capped initiator for the LAP of acrylate and methacrylate since its bulk structure and stable electron cloud can inhibit the carbanion from attacking the carbonyl group of these monomers [12,23,24]. Similarly, the presence of PSD species can hinder the side reactions between alkoxysilyl groups and active species. In addition, the sterically bulky DPE structure cannot be homopolymerized by any mechanism, except under extreme conditions [25]. Therefore, DPE derivatives have been used as co-monomers to regulate the polymer sequence through kinetic control [26,27,28].

Hence, in this work, DPE-SiOEt, a combination of the DPE structure and alkoxysilyl groups, was designed to hinder the side reactions between the alkoxysilyl groups and the active species and enable the preparation of in-chain sequence-controllable alkoxysilyl-functionalized polymers.

### 3.1. Synthesis of DPE-SiOEt

**Figure 1:**
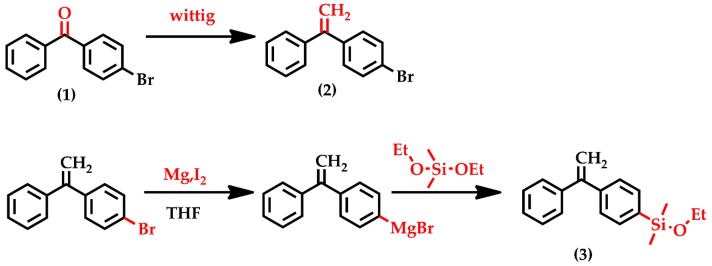
Synthetic route for 1-(ethoxydimethylsilyphenyl)-1-phenylethylene (DPE-SiOEt). (**1**) 4-bromobenzophenone; (**2**) 1-(4-bromophenyl)-1-phenylethylene (DPE-Br); (**3**) DPE-SiOEt.

The designed alkoxysilyl functionalized DPE derivative 1-(ethoxydimethylsilyphenyl)-1-phenylethylene (DPE-SiOEt) was synthesized using the Wittig reaction and then the Grignard reaction, as shown in Figure 1. Compound (2), 1-(4-bromophenyl)-1-phenylethylene, was prepared from (1), 4-bromobenzophenone, in high efficiency and 94% yield using the Wittig reaction. Before reacting it with MgI_2_, DPE-Br must be stirred and degassed over CaH_2_ to remove H_2_O and O_2_. Compound (3), 1-(ethoxydimethylsilyphenyl)-1-phenylethylene, was synthesized by adding DPE-MgBr dropwise into five equivalents of diethoxydimethylsilane. Providing the addition is slow, the reaction proceeds efficiently and affords the product in a yield of 50%. Further purification involved stirring and degassing over 5 wt % *n*-BuLi for at least 1 h, followed by distillation.

**Figure 2:**
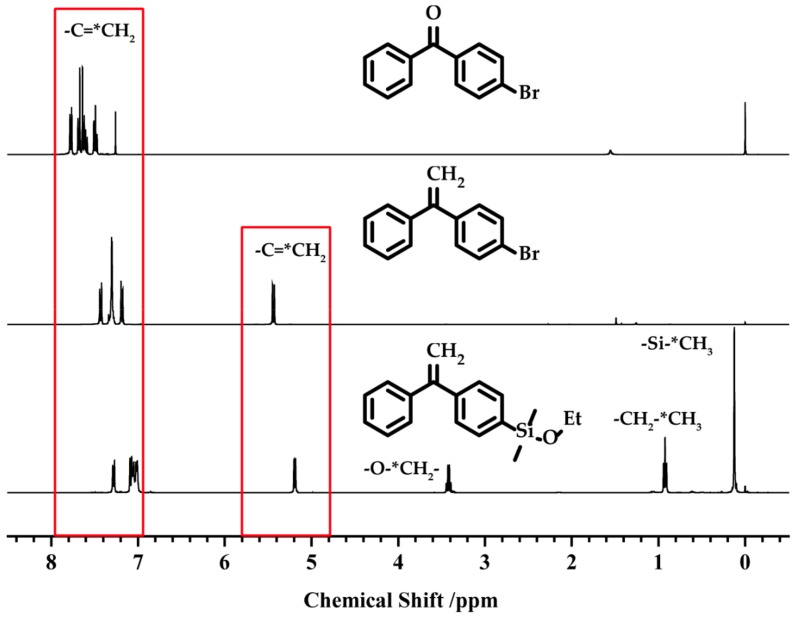
^1^H NMR spectra of (**1**–**3**). (**1**) 4-bromobenzophenone; (**2**) 1-(4-bromophenyl)-1-phenylethylene (DPE-Br); (**3**) 1-(ethoxydimethylsilyphenyl)-1-phenylethylene (DPE-SiOEt).

Reagents (1–3) were then characterized by ^1^H NMR (Figure 2). The appearance of a peak around δ = 5.5 ppm confirms the successful reaction between (1) and the Wittig reagent, and the appearance of peaks around δ = 3.4 and 0.9 ppm proved that DPE-MgBr was connected to diethoxydimethylsilane. The ratios of the areas of the peaks, from high field to low field, are 6:3:2:2:9, which is in good agreement with the theoretical value.

**Figure 3:**
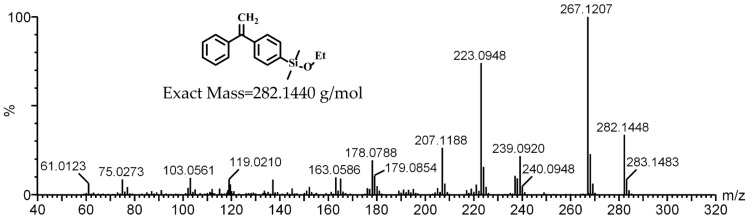
Electrospray ionization mass (EI-MS) spectrum of (3) DPE-SiOEt.

The novel monomer of DPE-SiOEt was also analyzed by electrospray ionization mass spectroscopy (EI-MS), as shown in Figure 3. EI-MS showed a mass at 282.1448 g/mol, which is consistent with the mass of the molecular formula C_18_H_22_SiO (theoretical value of 282.1440 g/mol). This characterization verifies that DPE-SiOEt was synthesized successfully. Furthermore, this synthetic route may provide a method for preparing various alkoxysilyl-functionalized DPE derivatives by substituting diethoxydimethylsilane with diisopropoxydimethylsilane, tetramethyl orthosilicate, etc.

### 3.2. Side Reaction between Alkoxysilyl Groups and Active Center during LAP

In 1950, silyl ethers were reported to be nucleophilically cleaved by organolithiums, as shown in Equation (1) [29]

(1) $$\mathrm{Si}-\mathrm{OR}+R^{\prime }Li\to Si-R^{\prime }+\mathrm{RO}-\mathrm{Li}$$

**Figure 4:**
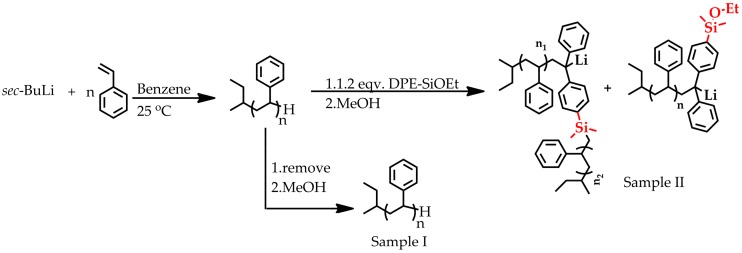
End-capping poly(styryl)lithium (PSLi) with DPE-SiOEt. First, sample I is removed from the solution. Then, PSLi is end-capped with DPE-SiOEt to afford sample II. (Design *M*_n_ = 2 kg/mol).

**Figure 5:**
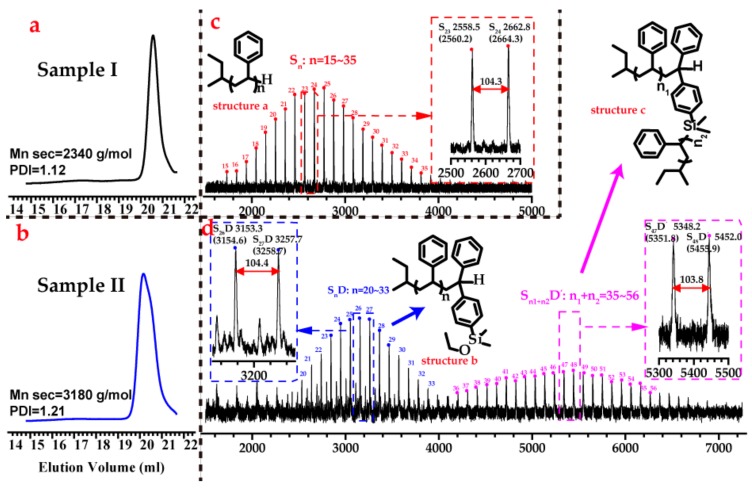
(**a**,**b**) Size exclusion chromatography (SEC) chromatograms of samples I and II. (**c**,**d**) MALDI-TOF spectra of samples I and II. The scheme of red peaks shows structure (**a**) homopolystyrene; the scheme of blue peaks shows structure (**b**) end-capped polystyrene; and the scheme of the pink peaks shows structure (**c**) the dimer structure.

Considering this study, Hirao et al. attempted to eliminate a side reaction in the homopolymerization of styrene derivatives bearing alkoxysilyl groups by lowering the polymerization temperature to −78 °C. Interestingly, when adding PSLi to a solution of this type of functionalized homopolystyrene, they observed the formation of a grafted structure [30,31]. Because PSLi undoubtedly can cleave the alkoxysilyl groups, we end-capped PSLi with DPE-SiOEt to decrease or even eliminate the side reaction between the alkoxysilyl groups and the active center. As shown in Figure 4, styrene was first initiated by *sec*-BuLi at 25 °C in benzene, and the resulting PSLi, with a designed molecular weight of 2 kg/mol, was prepared. After removing sample I from the solution, the DPE-SiOEt (DPE-SiOEt/Li = 1.2) was added to end-cap the polymer chains. Both sample I and sample II were characterized by SEC and MALDI-TOF-MS, and the relevant results are shown in Figure 5.

The single peak in the SEC chromatogram of sample I, as shown in Figure 5a, demonstrates the narrow polydispersity of the polymer, suggesting that no side reactions occur during the homopolymerization of styrene. Regarding the SEC chromatogram in Figure 5b, the peak moves a little forward, and an obvious shoulder peak appears. DPE-SiOEt seems to function as a coupling agent in the system, although some of the chains are end-capped with DPE-SiOEt.

MALDI-TOF-MS is widely used for the determination of polymer sequences because it quantifies the exact molecular weight of all chains in a sample [26,32], enabling the composition, end groups and structure of the sample to be calculated. Therefore, the samples were further characterized by MALDI-TOF-MS. The peak with a *m*/*z* of 2558.5 in Figure 5c corresponds to 23 units of styrene (23 × 104.1 u) + the hydrogen end-group (1.0 u) + the counterion Ag (107.9 u) + the *sec*-butyl end-group (57.0 u), which are attributed to the structure S_23_. Similarly, the peaks marked in red are attributed to the structures in the following domain: S_n_, *n* = 15~35. These spectra showed that the linear polystyrene without side-reaction was first prepared.

The two distributions shown in Figure 5d are colored blue and pink. The main peak of the blue distribution, which has a *m*/*z* of 3153.7, corresponds to 26 units of styrene (26 × 104.1 u) + the DPE-SiOEt unit (282.1 u) + the hydrogen end-group (1.0 u) + the counterion Ag (107.9 u) + the *sec*-butyl end-group (57.0 u). The peaks marked in blue are attributed to the structures in the domain: S_n_D, *n* = 20~33. The other distribution marked in pink is attributed to the structures in the domain: S_n1+n2_D’, *n*1 + *n*2 = 35~56. The most intense peak in this distribution, which has an *m*/*z* of 5348.2, corresponds to 47 units of styrene (47 × 104.1 u) + the DPE-SiOEt unit (282.1 u) + the counterion Ag (107.9 u) + two *sec*-Butyl end-groups (2 × 57.1 u)—the missing OEt group from DPE-SiOEt (−45.1 u).

Compared to the red distribution, the blue distribution confirmed that some of the chains are end-capped by DPE-SiOEt, as shown in structure b in Figure 5. However, the pink peaks in Figure 5 show that the side reaction still occurred and doubled the molecular weights by forming a dimer structure, as shown in structure c in Figure 5, indicating that the active center reacts with the Si atom and substitutes the OEt group. Interestingly, the pink peaks also suggest that there is only one DPE-SiOEt unit attached to the chains; no signals in those peaks correspond to two DPE units, indicating that the living species with DPE ends did not participate in the side coupling reaction. Once the DPE structures end-cap the polystyrene chain, such living species might hardly react with the alkoxysilyl groups, and the side reaction can only occur between styrene-end living center alkoxysilyl groups. In particular, this result might indicate that the alkoxysilyl groups react with PSLi, but not with PSDLi.

### 3.3. Kinetic Control of the Side Coupling Reaction

According to the result described above, the side reaction is related to [D^−^] and [S^−^], where [D^−^] and [S^−^] are the concentrations of a reactive center with a DPE unit and a styrene unit end-cap respectively. Because PS^−^ and PSD^−^ species are always present at the same time in the copolymerizations of styrene and DPE derivatives, PS^−^ can react with and cleave the alkoxysilyl groups. Decreasing the concentration of PS^−^ and increasing the concentration of PSD^−^ might be a facile strategy to eliminate the side reaction.

**Figure 6:**
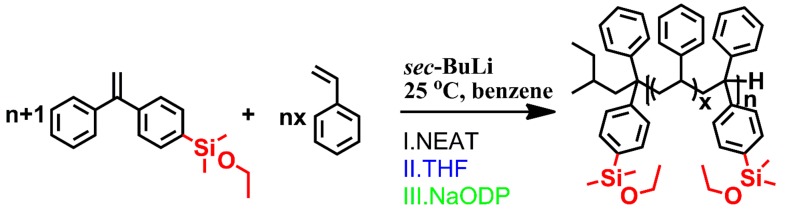
The copolymerization of DPE-SiOEt and styrene initiated by *sec*-BuLi in benzene at 25 °C.

Considering this strategy, a series of copolymerizations with different feed ratios of DPE-SiOEt to styrene were conducted in the glove box. Furthermore, additional copolymerizations with THF or NaODP as additives were also carried out to investigate the mechanism of the side reaction. These reactions were initiated using *sec*-BuLi in benzene at 25 °C, as shown in Figure 6. The polymerizations lasted 24 h for the exhaustion of styrene. In addition, the colorless, transparent solution of the monomers turned dark red immediately as soon as the stoichiometric *sec*-BuLi was added. In addition to this, the color remained and hardly faded over 7 days.

All samples were characterized by ^1^H NMR and SEC. The composition and structure of the copolymers synthesized under neat conditions are shown in Table 1.

**Figure 7:**
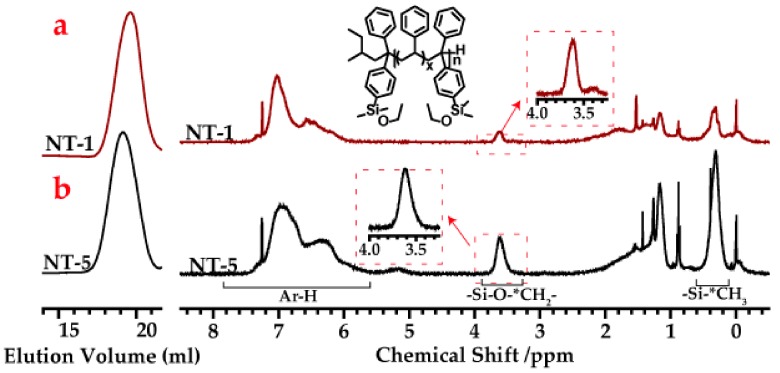
SEC chromatograms and ^1^H NMR spectra of sample NT-1 (**a**) and sample NT-5 (**b**).

The ^1^H-NMR and SEC spectra of NT-1~NT-9 are shown in Figure S1. Here, we chose two of them to give a brief description. As seen in Figure 7, the simultaneous complete disappearance of the peak at approximately 5.5 ppm and the appearance of the peak at approximately 3.6 ppm indicate the occurrence of the crossover reaction because of the nucleophilic attack of PSLi on the double bond of DPE-SiOEt. In addition, the weak broad peak at approximately 5.3 ppm is ascribed to the macroblock of styrene.

The composition of the samples ([*M*_S_]/[*M*_D_]) were calculated from the ^1^H NMR and SEC spectra using Equation (S1) in the ESI. The average number of alkoxysilyl functional groups in each polymer chain was calculated using both Equation (S1) and Equation (S2). Area (aromatic region) is the integrated area from δ = 5.0 to δ = 7.5 ppm after subtracting the area of the CDCl_3_ region (δ = 7.25–7.30 ppm). Area (–OCH_2_–) is the integrated area from δ = 3.2 ppm to δ = 3.8 ppm. The compositions obtained are listed in Table 1, which proves that a novel type of in-chain alkoxysilyl functionalized polystyrene was prepared.

**Figure 8:**
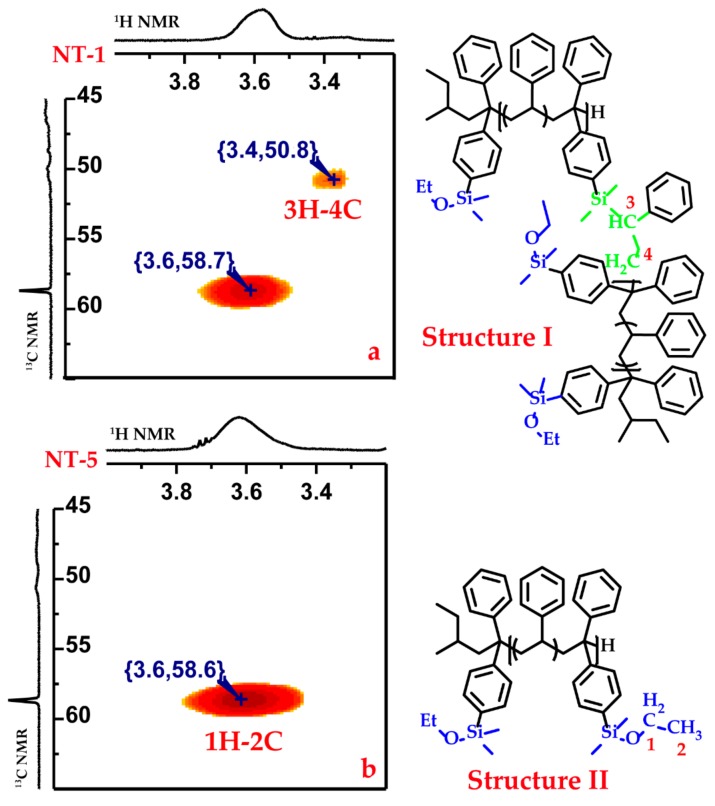
(**a**,**b**) ^1^H-^13^C HMBC 2D NMR spectra of NT-1 and NT-5. Structure (**I**) is probably formed by the side reaction, whereas structure (**II**) corresponds to the in-chain functionalized polystyrene.

However, as shown in Table 1, the PDI decreases slightly as the feed ratio of styrene to DPE-SiOEt ([*M*_S_]_0_/[*M*_D_]_0_) decreases. Figure 7 shows that the single peak for NT-5 became two as the feed ratio of styrene to DPE-SiOEt increased, suggesting that the side reaction can be controlled by adding more DPE-SiOEt into the polymerization. To test this hypothesis, ^1^H-^13^C HMBC 2D NMR was applied to analyze the structures of the copolymers. The signal response of both samples at approximately (3.6, 58.6), as shown in the 2D NMR spectra in Figure 8a,b, is because of the heteronuclear long-range correlation of 1H-2C in the alkoxysilyl group, as shown in structure I in Figure 8. The signal response at (3.4, 50.6) may arise from the correlation of 3H-4C in the structure formed by the side reaction, as shown in structure II in Figure 8. Because no signal response of 3H-4C was observed for sample NT-5, the corresponding polymerization was stable and did not lead to the formation of new structures. The correlation signal response of 3H-4C becomes obvious as the St/DPE-SiOEt feed ratio is increased to 8/1 (NT-1, Figure 8a), a result that might verify that the side reaction can be eliminated when [*M*_S_]_0_/[*M*_D_]_0_ is less than one.

The reactivity ratio, which is determined by the Mayo-Lewis Equation (S3), is an essential index that holds great importance for estimating the copolymerization tendency of two monomers. The Mayo-Lewis equation is suitable for situations in which the styrene monomer is consumed and no side reaction occurs [26]. For DPE derivatives with *r*_St_ values less than or close to 1, such as dimethyl-(4-(1-phenylvinyl)phenyl)silane (DPE-SiH, *r*_St_ = 0.38), the DPE and styrene units are introduced into chains one by one at the beginning of the copolymerization, and then styrene continues to homopolymerize once DPE is consumed. However, if the molar feed ratio (St/DPE) is larger than one, the homopolystyrene block will emerge at the end of the chain, and the composition will eventually be equal to the feed ratio because of the property of living species. Furthermore, when the copolymerization is terminated in advance, we cannot obtain the concentration of styrene, and so the instantaneous molar feed ratio also cannot be calculated. For these reasons, accurate reactive ratios for styrene in the copolymerization of DPE derivatives whose *r*_St_ is close to or less than one have only been calculated when the molar feed ratio of DPE was equimolar or in excess of the styrene.

The average reactive ratio for styrene in the copolymerization of DPE-SiOEt is calculated as 1.5. We only gave the reactive ratios of NT-5~NT-9, as listed in Table 1, because NT-1~NT-4 were synthesized with high molar feed ratios (St/DPE larger than 1), which might lead to a deviation. The DPE unit’s arrangements of the copolymer are related to the reactive ratios. In former research, the sequence of the copolymer of styrene and DPE-SiH (*r*_St_ = 0.38 [33]) shows that the DPE-SiH prefer introduction onto chains first and so does DPE (*r*_St_ = 0.45) [34,35,36]. By contrast, DPE-(NMe_2_)_2_ (*r*_St_ = 54.9) [15] distribute at the end section of the whole polymer chains. DPE-SiH/NMe_2_(*r*_St_ = 1.29) [37], as reported, has a similar *r*_St_ value to DPE-SiOEt, and the corresponding sequence of the copolymer of styrene and DPE-SiH/NMe_2_ was determined as the relatively homogeneous distribution of DPE units, separated by an average of four styrene units.

In addition, *r*_St_ generally obeys the Hammett relationship and is described by Equation (S4) [34]. The value of σ for the *p*-ethoxydimethylsilyl substituent is calculated as −0.30, implying that the ethoxydimethylsilyl substituent is a weak electron-donating group.

To further investigate the polymerizations of DPE-SiOEt and styrene, the effect of THF (THF/Li = 10) and NaODP (NaODP/Li = 2) on them were studied. The composition and structure results are listed in Table 2, and their ^1^H NMR spectra are shown in Figure S2.

**Figure 9:**
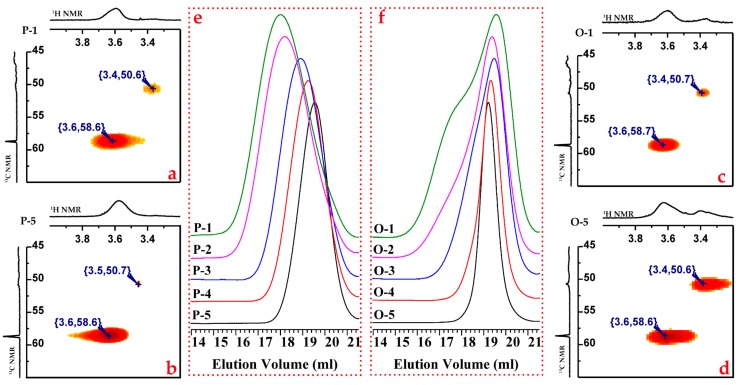
(**a**–**d**) The ^1^H-^13^C HMBC 2D NMR spectra of P-1, P-5, O-1, and O-5, respectively. (**e**,**f**) The SEC chromatograms of P-1 and P-5.

As shown in Figure 9e, samples P-5 to P-1, products of the copolymerization of styrene and DPE-SiOEt with a designed molecular weight of approximately 10 kg/mol, increased in molecular weight, and molecular weight distributions increased as the molar feed ratio (St/DPE) increased. These results suggest that a minor side reaction led to a decrease in active center concentration. The same phenomenon occurs in the presence of NaODP. Although the molecular weights changed little between O-5 and O-1, the molecular weight distribution of the samples increased considerably. In particular, a bimodal distribution is observed in Figure 9f for O-1, which has the 8/1 molar feed ratio (St/DPE).

THF and NaODP were tested as additives because of their potentially different results. Adding THF could increase the styrene polymerization reaction rate by increasing the concentration of unassociated species (P-Li) (THF) [38,39]. Thus, the living center might first consume the styrene and then react with the alkoxysilyl groups, forming the dimeric polymers with a higher molecular weight. NaODP might form an [(P,Li)OR]Na species [39,40], thus increasing the interionic distance. This species is highly likely to increase both the styrene and DPE-SiOEt polymerization rate, besides, they could also increase the probability of reacting with the alkoxysilyl groups. Unfortunately, the 2D NMR spectra of all these samples (P-1, P-5 O-1, and O-5) have a signal response at (3.4, 50.6), indicating that the side reaction occurred.

These results lead to the conclusion that the side reaction becomes predominant when THF and NaODP were added to the copolymerization and, similarly, that the side reaction was inhibited by decreasing the feed ratio of styrene to DPE-SiOEt.

## 4. Conclusions

In conclusion, in-chain alkoxysilyl functionalized polystyrene was synthesized at 25 °C in benzene via LAP using a novel DPE derivative—DPE-SiOEt—as a co-monomer. Compared to styrene derivatives with alkoxysilyl groups, this DPE derivative inhibited the side coupling reaction between the alkoxysilyl groups and the reactive center. However, MALDI-TOF-MS demonstrated that this side reaction still occurred when DPE-SiOEt was used to end-cap PSLi. By contrast, PSDLi did not react with the alkoxysilyl groups because of the steric hindrance and the electron cloud stabilization of the DPE structure; therefore, the alkoxysilyl groups are only cleaved by PSLi. The mechanism was evidenced by the kinetic control of the feed ratio in the copolymerization of styrene and DPE-SiOEt. As the concentration of [PS-] was decreased, the side reaction was controlled in a stable way. In addition, the copolymerization of styrene and DPE-SiOEt has an *r*_St_ of 1.5.

## Figures and Tables

**Table 1:** Composition, structures and reactive ratios for copolymers of styrene and DPE-SiOEt in the absence of additives.

No. ^a^	[*M*_S_]_0_/[*M*_D_]_0_^b^	[*M*_S_]/[*M*_D_] ^c^	*M*_n_^d^	PDI ^d^	N_D_^e^	*r*_St_^f^
	(mol/mol)	(mol/mol)	(kg/mol)			
NT-1	8:1.0	6.03	11.6	1.40	12.8	N/A
NT-2	6:1.0	4.68	9.6	1.39	12.5	N/A
NT-3	4:1.0	3.85	10.3	1.38	15.0	N/A
NT-4	2:1.0	2.31	12.0	1.32	23.0	N/A
NT-5	1:1.0	1.72	12.8	1.30	25.5	1.3
NT-6	1:1.2	1.64	11.4	1.33	25.2	1.5
NT-7	1:1.4	1.56	10.6	1.33	23.9	1.5
NT-8	1:1.6	1.59	10.2	1.33	22.8	1.9
NT-9	1:1.8	1.43	11.2	1.32	26.0	1.5

^a^ All samples were synthesized at 25 °C for 24 h, the designed *M*_n_ is 10 kg/mol; ^b^ The monomer molar feed ratio of styrene and DPE-SiOEt; ^c^ The ratios of the two monomer units in the final copolymer, calculated from the ^1^H NMR spectra using Equation (S1); ^d^ Molecular weight and molecular weight distributions were determined using SEC calibrated with polystyrene standards; ^e^ The average number of 1,1-diphenylethylene (DPE) in each chain, calculated from the ^1^H NMR spectra and SEC data using both Equation (S1) and Equation (S2); ^f^ N/A means that the calculation method for reactive ratio shown below is not applicable to this sample.

**Table 2:** Composition and structure results for the copolymerization of styrene and DPE-SiOEt in the presence of the additive.

No. ^a^	Additive	[*M*_S_]_0_/[*M*_D_]_0_^b^	[*M*_S_]/[*M*_D_] ^c^	*M*_n_^d^	PDI ^d^	*N*_D_^e^
		(mol/mol)	(mol/mol)	(kg/mol)		
P-1	THF/Li = 10	8.0	9.32	22.0	1.49	17.7
P-2		6.0	6.41	20.7	1.42	21.9
P-3		4.0	4.87	13.9	1.39	17.6
P-4		2.0	2.63	11.7	1.36	21.1
P-5		1.0	2.14	9.6	1.31	19.2
O-1	NaODP/Li = 2	8.0	5.62	13.7	1.83	15.8
O-2		6.0	4.73	14.0	1.81	18.3
O-3		4.0	3.41	12.5	1.44	19.7
O-4		2.0	1.66	12.6	1.25	28.2
O-5		1.0	1.08	13.2	1.20	32.9

^a^ All samples were synthesized at 25 °C for 24 h, designed *M*_n_ is 10 kg/mol.; ^b^ The monomer molar feed ratio of styrene and DPE-SiOEt.; ^c^ The ratios of the two monomer units in the final copolymer, calculated from the 1H NMR spectra using Equation (S1); ^d^ Molecular weights and its distributions were determined by SEC using polystyrene standards for calibration.; ^e^ The average number of DPE in each chain, calculated from the ^1^H NMR spectra and SEC data using both Equation (S1) and Equation (S2).

## Supplementary Materials

The following are available online at www.mdpi.com/2073-4360/9/5/171/s1, Figure S1: The SEC and ^1^H NMR spectra of NT-1~NT-9, Figure S2: The 1H NMR spectra of O-1~O-5 and P-1~P-5, Equations (S1) and (S2): the calculation of the in-chain functionalized alkoxysilyl copolymer composition, Equations (S3)~(S5): the calculation of reactivity ratio of the copolymerization for the styrene and DPE-SiOEt.
