# Supplementary material for: Study on the Mechanism of a Side Coupling Reaction during the Living Anionic Copolymerization of Styrene and 1-(Ethoxydimethylsilyphenyl)-1-phenylethylene (DPE-SiOEt)
Source: Polymers (Basel). 2017 May 11;9(5):171. doi: 10.3390/polym9050171 (PMC6432263; doi:10.3390/polym9050171)
Supplement: Supplementary file 1 [file polymers-09-00171-s001.pdf]

Supporting Information: Study on the Mechanism of a Side Coupling Reaction during the Living Anionic Copolymerization of Styrene and 1-(Ethoxydimethylsilyphenyl)-1-phenylethylene (DPE-SiOEt)

Pibo Liu, Hongwei Ma, Heyu Shen, Li Han, Shuang Chang, Long Zang, Yiyu Bian, Yu Bai and Yang Li

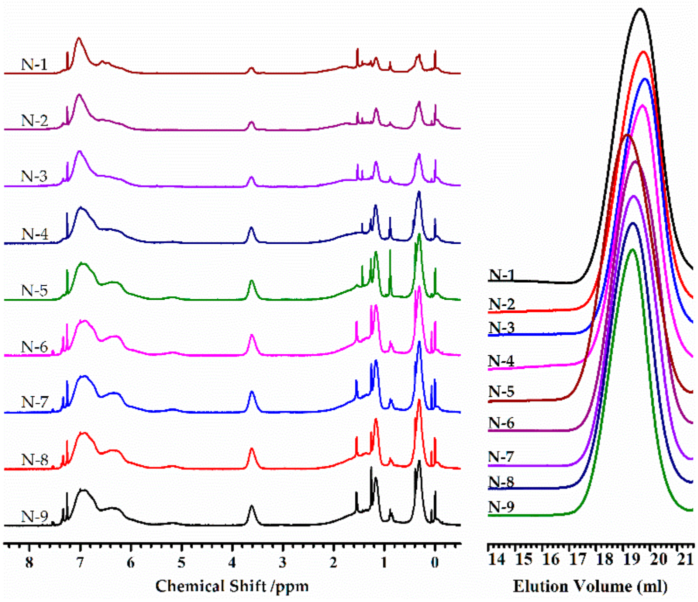

Figure S1. The SEC and ^1H NMR spectra of NT-1~NT-9.

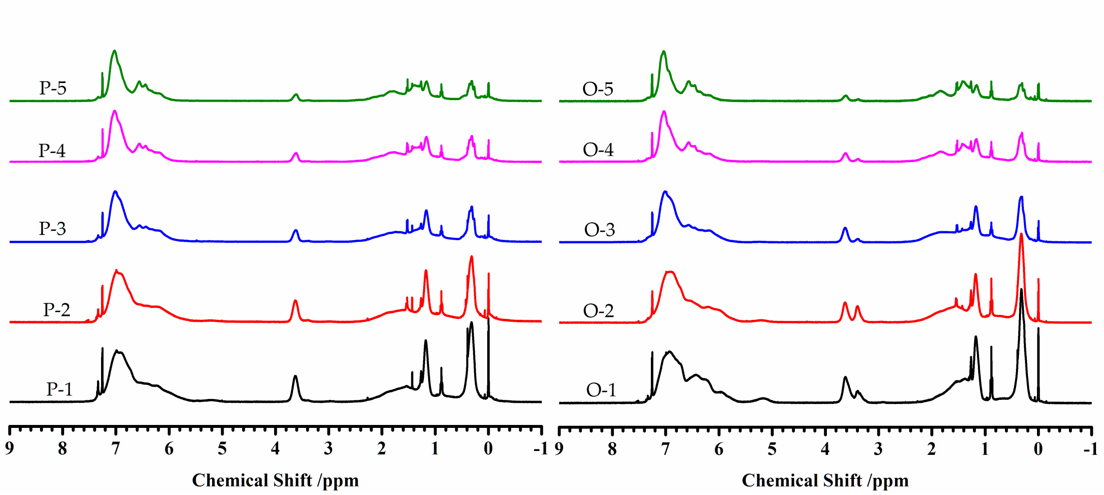

Figure S2. The ^1H NMR spectra of O-1~O-5 and P-1~P-5.

Equations:

$$\frac{2N_D}{5N_S+9N_D} = \frac{Area(-OCH_2-)}{Area(aromatic\ region)} \quad \text{(S1)}$$

$$M_S N_S + M_D N_D = \overline{M_n} \quad \text{(S2)}$$

where N_D refers to the average number of DPE-SiOEt units incorporated into the copolymer. M_D, M_S and $\overline{M_n}$ are the molecular weights of DPE-SiOEt, styrene and the copolymer, respectively.

$$\ln\frac{[M_D]}{[M_D]_0}+\frac{1}{r_{St}-1}\ln\left\{\frac{[M_S]_0}{[M_D]_0}(r_{St}-1)+1\right\}=0 \tag{S3}$$

$$\lg\frac{r_{St}^0}{r_{St}}=\rho\sigma \tag{S4}$$

where r_{St}^0 is the reactivity ratio of the copolymerization for the styrene and DPE (r_{St}^0=0.45) in benzene[1]. ϱ=+1.8[2,3] is substituted into Equation (S4).

1. Yamagishi, A.; Szwarc, M. Kinetics of styrene addition in benzene solution to living lithium polymers terminated by 1,1-diphenylethylene units. The effect of mixed dimerization of monomeric polymers. *Macromolecules* **1978**, *11*, 504-506.
2. Quirk, R.P.; Lee, B. Experimental criteria for living polymerization. *Polym. Int.* **1992**, *27*, 359-367.
3. Wu, L.L.; Wang, Y.S.; Wang, Y.R.; Shen, K.H.; Li, Y. In-chain multi-functionalized polystyrene by living anionic copolymerization with 1,1-bis(4-dimethylaminophenyl)ethylene: Synthesis and effect on the dispersity of carbon black in polymer-based composites. *Polymer* **2013**, *54*, 2958-2965.
